# Supplementary material for: Tailoring atomic diffusion for in situ fabrication of different heterostructures
Source: Nat Commun. 2021 Aug 10;12:4812. doi: 10.1038/s41467-021-25194-2 (PMC8355324; doi:10.1038/s41467-021-25194-2)
Supplement: Supplementary file 1 — Supplementary Information [file 41467_2021_25194_MOESM1_ESM.pdf]

SUPPLEMENTARY INFORMATION

Tailoring atomic diffusion for in situ fabrication of different heterostructures

Hui Zhang[1], Tao Xu[1*], Kaihao Yu[1], Wen Wang[1], Longbing He[1] & Litao Sun[1,2*]

[1] SEU-FEI Nano-Pico Center, Key Laboratory of MEMS of Ministry of Education, School of Electronic Science and Engineering, Southeast University, Nanjing 210096, China.

[2] Center for Advanced Materials and Manufacture, Joint Research Institute of Southeast University and Monash University, Suzhou 215123, China.

Correspondence and requests for materials should be addressed to Tao Xu (email: xt@seu.edu.cn) or to Litao Sun (email: slt@seu.edu.cn).

Supplementary Figures

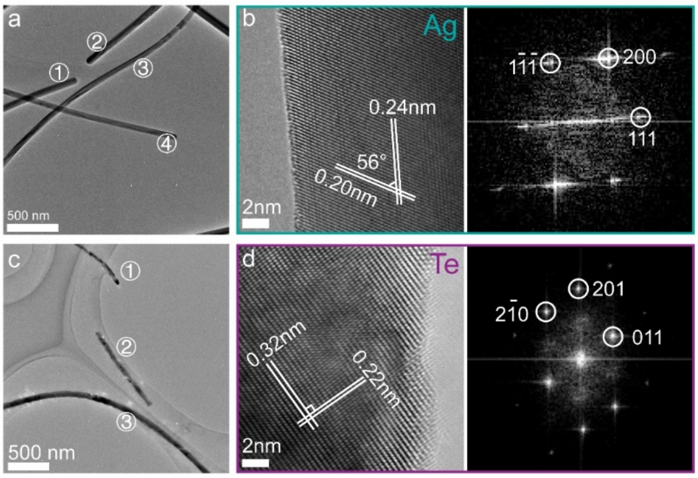

Supplementary Figure 1. TEM images of Ag nanowires and Te nanowires. a TEM image of 4 Ag nanowires, with different diameters of 72, 78, 68 and 65 nm (①, ②, ③ and ④, respectively). **b** HRTEM image and the corresponding FFT pattern of Ag nanowire. **c** TEM image of 3 Te nanowires, with different diameters of 56, 67 and 77 nm (①, ② and ③, respectively). **d** HRTEM image and the corresponding FFT pattern of a Te nanowire.

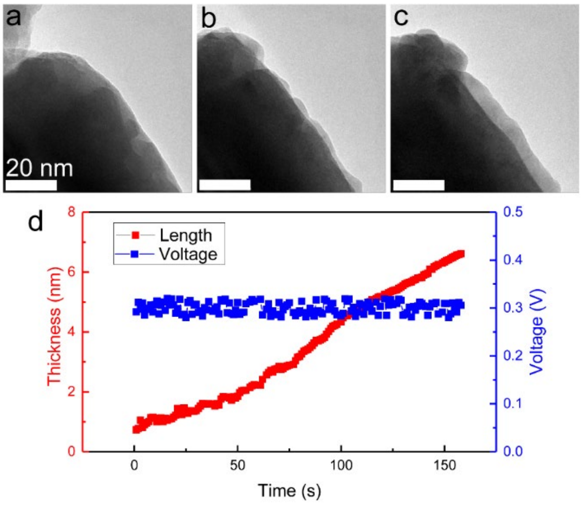

Supplementary Figure 2. The thickening process of Ag2Te shell after applying bias in TEM. a-c TEM images of the growth process showing coalescence of Ag_2Te hillocks and thickening. **d** The thickness of Ag_2Te shell varies with the time under a bias. The bias applied at Ag nanowire is kept stable at 0.3 V. The growth of Ag_2Te shell is near-linear with time (0.03988 nm/s).

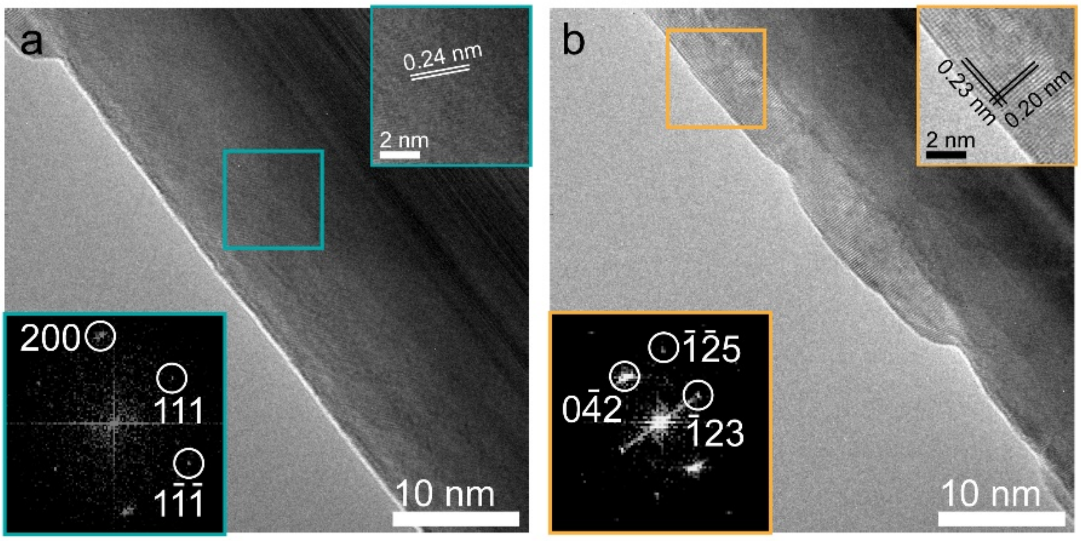

Supplementary Figure 3. Detailed characterization of Ag and Ag_2Te crystallite. The insets show HRTEM images and the corresponding FFT patterns of **a** Ag and **b** Ag_2Te respectively. The orientation relationship of {111} Ag (2.30Å) and {$\bar{1}$23} Ag_2Te (2.36Å) can be identified with the HRTEM and FFT images.

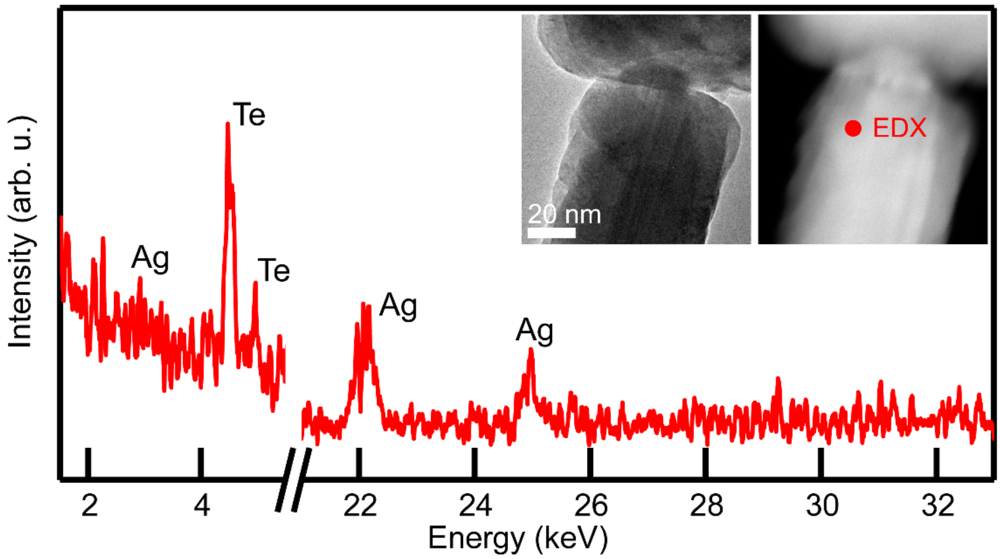

Supplementary Figure 4. The EDX analysis of Ag_2Te shell covering the Ag nanowire. The insets show the TEM and HAADF images of the Ag_2Te-Ag core-shell structure. A strong Te signal can be captured at the front part of Ag nanowire, which means Te atoms has migrated into Ag nanowire. The atomic ratio of Ag to Te is (2.85 ± 0.57):1 as determined from a quantitative analysis of the spectrum. This atomic ratio is a little higher than that in Ag_2Te indicating that there is a significant amount of Ag in the core.

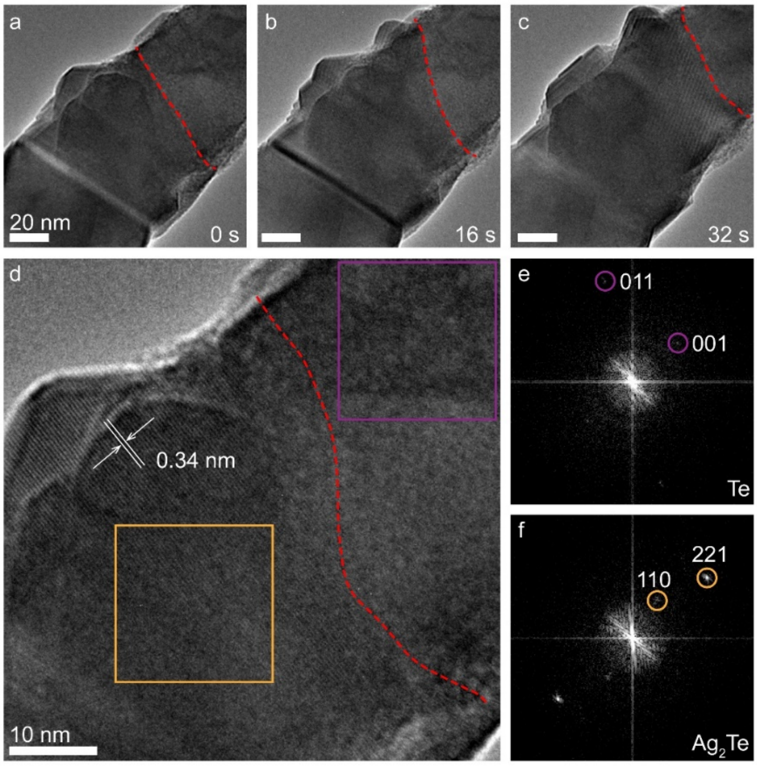

Supplementary Figure 5. HRTEM image series of the phase transformation frontier. a-c A series of in situ TEM micrographs showing the process of phase transition. **d** A HRTEM image of the reaction product; red dashed line indicates the phase transformation frontier. **e-f** FFT patterns transformed from different regions beside the frontier reveal the transformation from Te to Ag_2Te.

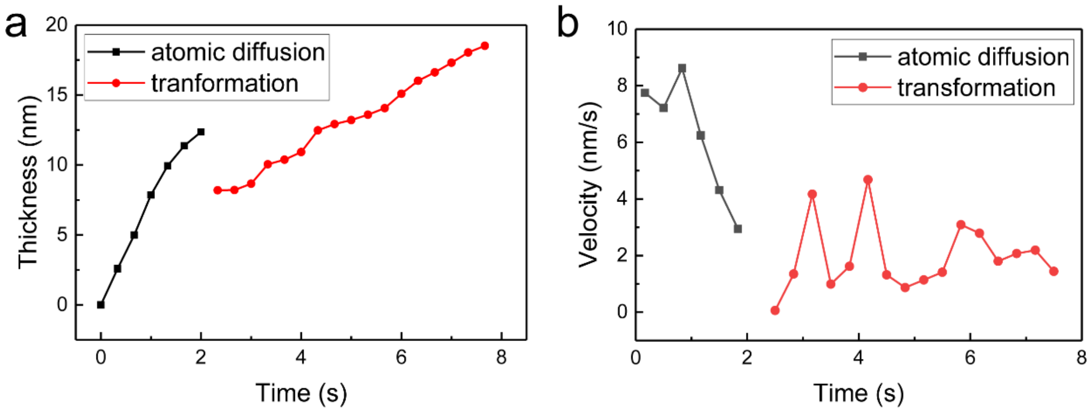

Supplementary Figure 6. The diffusion of Ag atoms and transformation of Ag_2Te crystallite with time. a The diffusion length of Ag atoms and growth of Ag_2Te. **b** The diffusion velocity and transformation velocity vary with time. The diffusion of Ag atoms slows down with time, while the transformation of Ag_2Te keeps stable with time.

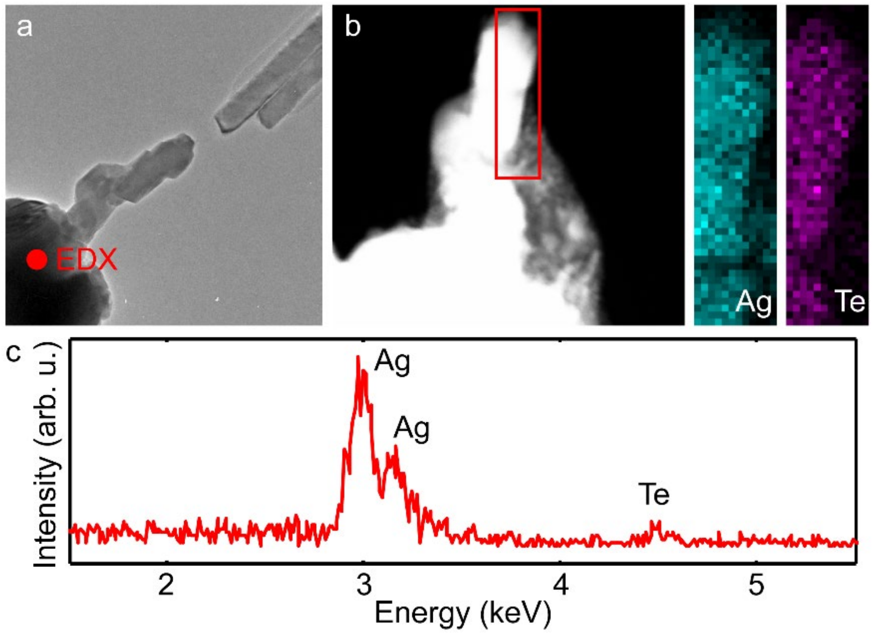

Supplementary Figure 7. HAADF image and EDX analysis of Ag_2Te-Te hetero-nanostructure. a Low magnification TEM image of Ag_2Te-Te structure with a break at the phase transformation frontier. **b** HAADF image and EDS mapping of the Ag_2Te part of the heterostructure. The atomic ration of Ag to Te obtained from quantitative analysis is (2.47 ± 0.39):1, which is a little larger than the ratio of Ag2Te; while that ratio of transition area is only (0.20 ± 0.03):1 (Fig. 4h). **c** EDX spectrum taken in the area marked by the of the red point of in (a). A weak single signal of Te element can be captured in the front point part of the Ag NW, though the atomic ratio of Te to Ag is only 1:(73.31 ± 35.27). This suggests that a bit of some thermal diffusion and concentration gradient diffusion are also existed take place in the system. However, the electrically-driven diffusion is that plays the most major role in causing the transformations.

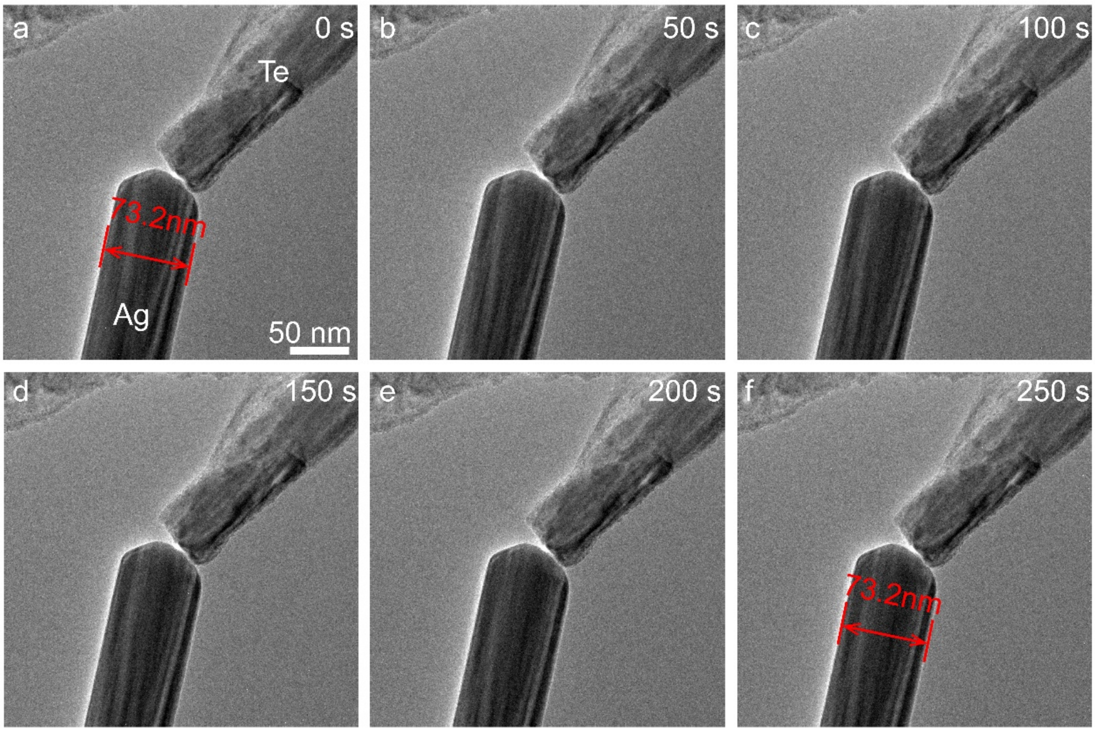

Supplementary Figure 8. In-situ experiments only under electron beam irradiation without voltage bias.

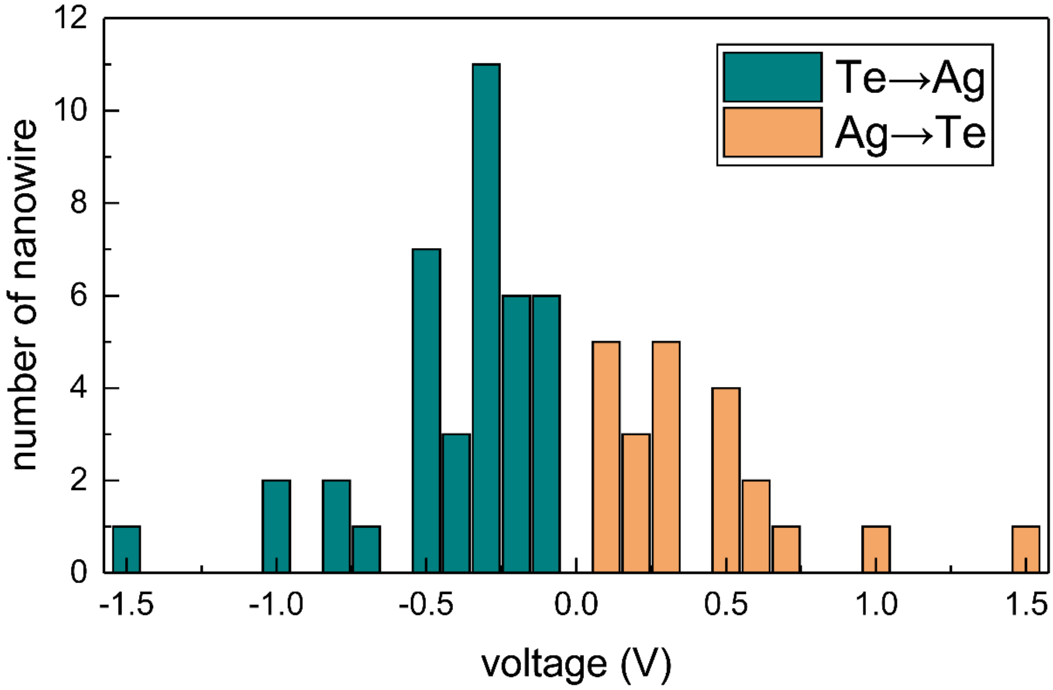

Supplementary Figure 9. Number of nanowires that were observed for each of the voltage conditions. The arrows show the direction of current.

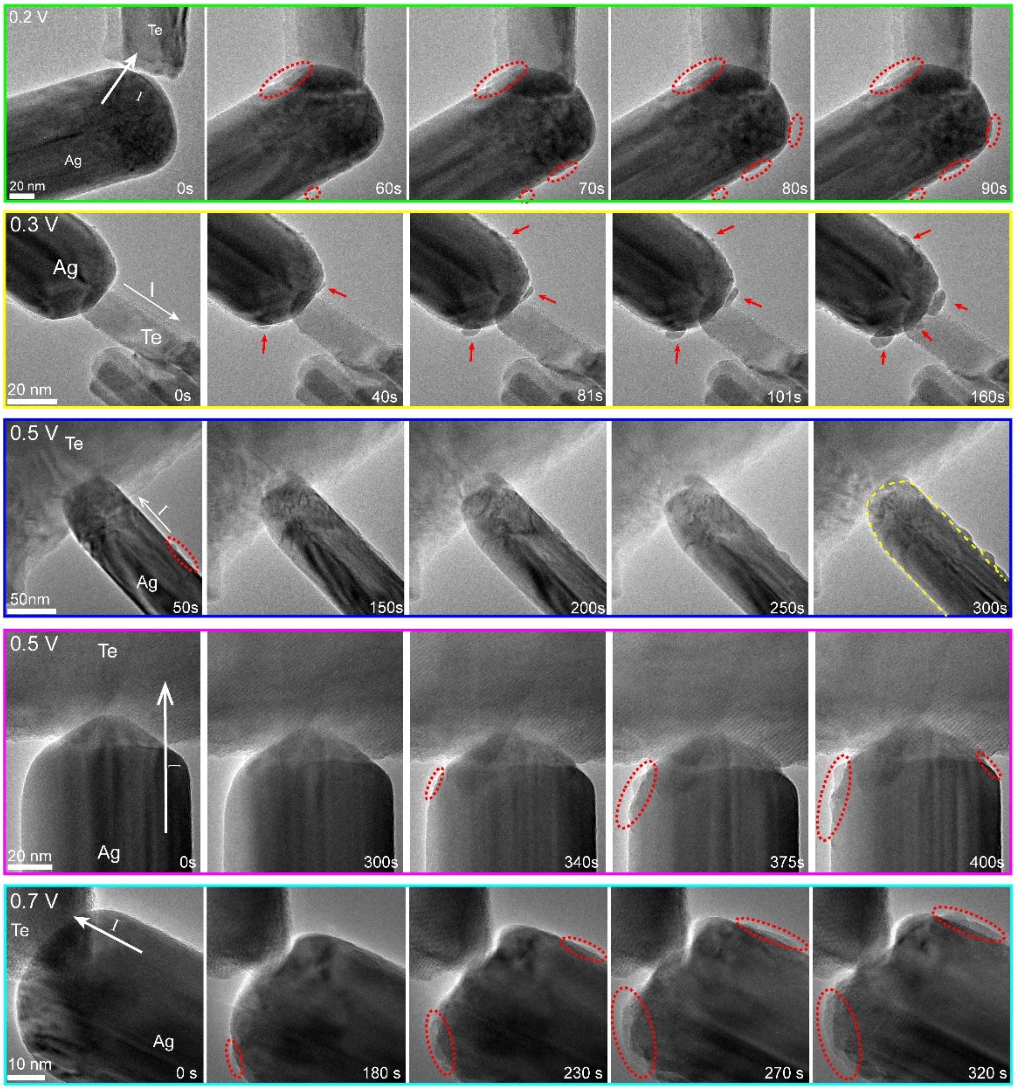

Supplementary Figure 10. Electrically driven diffusion of Te atoms under the bias of Ag to Te.

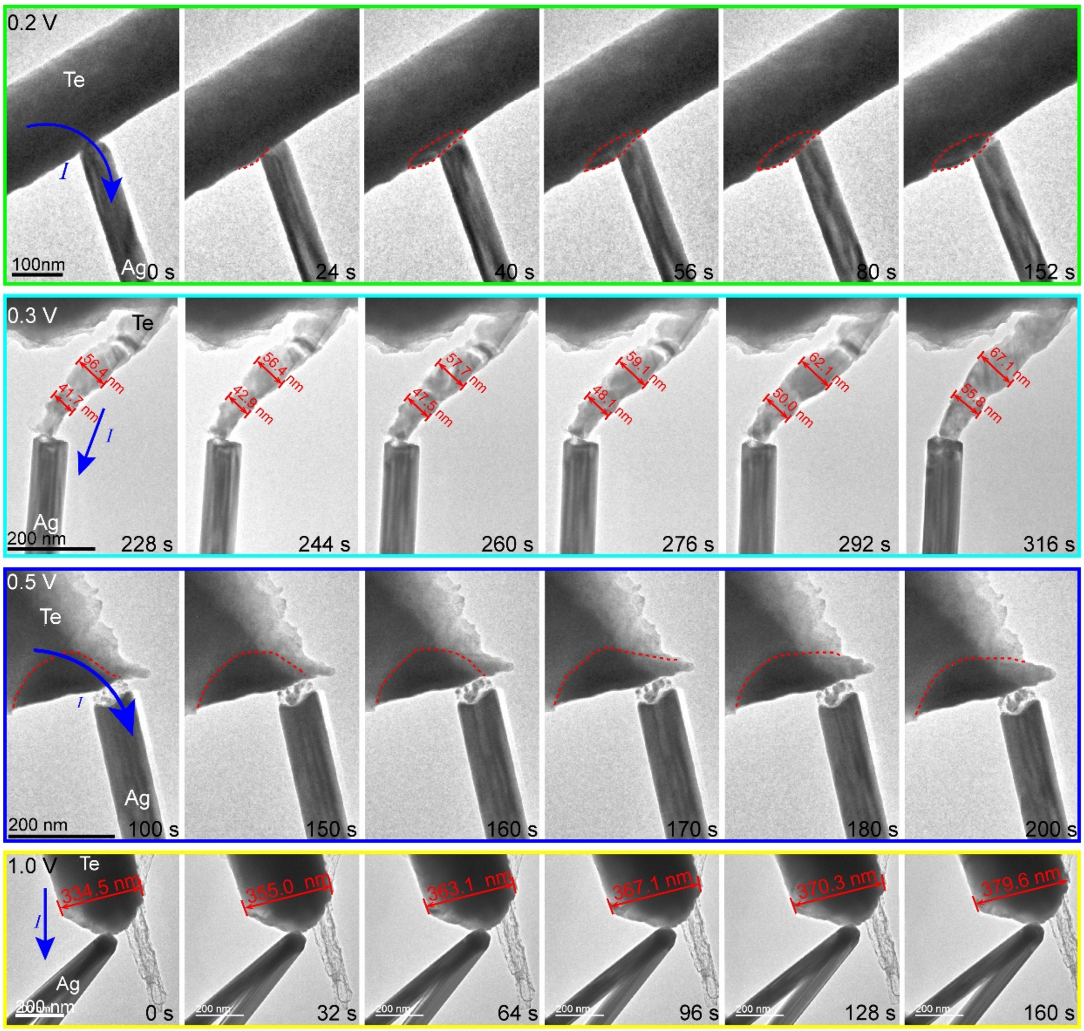

Supplementary Figure 11. Electrically driven diffusion of Ag atoms under different conditions.

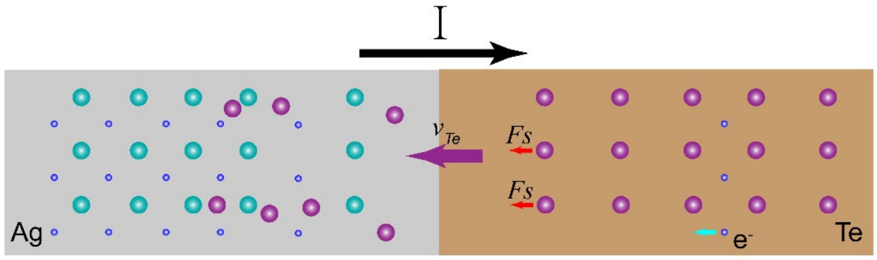

Supplementary Figure 12. Schematic illustration of forces acting on the Ag-Te system when current flows from the Ag nanowire to the Te nanowire. When an electric current passes through the material, collisions between the conduction electrons and the atoms or ions lead to drift of the ions. There are actually two forces acting on an ion, the force on the ions due to the presence of the electric field (electrostatic force, *Fi*), and the momentum transfer from the conduction particles (electron-wind force, *Fs*).

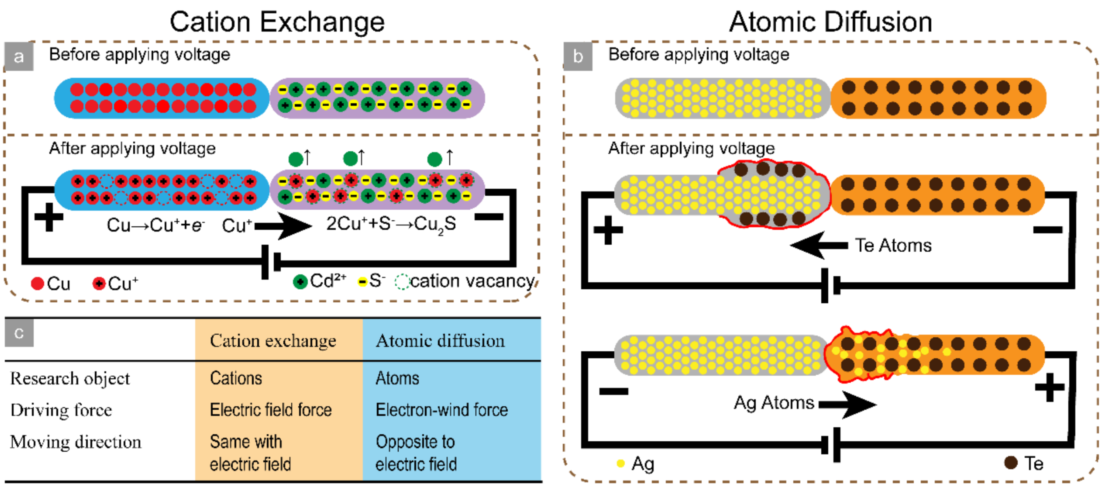

	Cation exchange	Atomic diffusion
Research object	Cations	Atoms
Driving force	Electric field force	Electron-wind force
Moving direction	Same with electric field	Opposite to electric field

Supplementary Figure 13. Schematic illustration showing the difference of the mechanisms between our previous work[1] and current work. a The mechanism of the previous work "electrically driven cation exchange". The Cu electrode was electro-ionized under the influence of a high voltage (larger than the standard electrode potential of Cu, 0.339V), after which Cu^+ migrated into the CdS nanorod along the direction of the electric field and reacted to form Cu_2S. **b** The mechanism of our present work "atomic diffusion driven by electron-wind force". A low voltage was applied to the Ag-Te system, as a result, Ag or Te atoms could not be ionized electrochemically. Regardless the direction of electric field, the Ag or Te atoms always diffuse opposite to the direction of electric field and then spontaneously react into Ag_2Te. **c** A summary of differences between the two works.

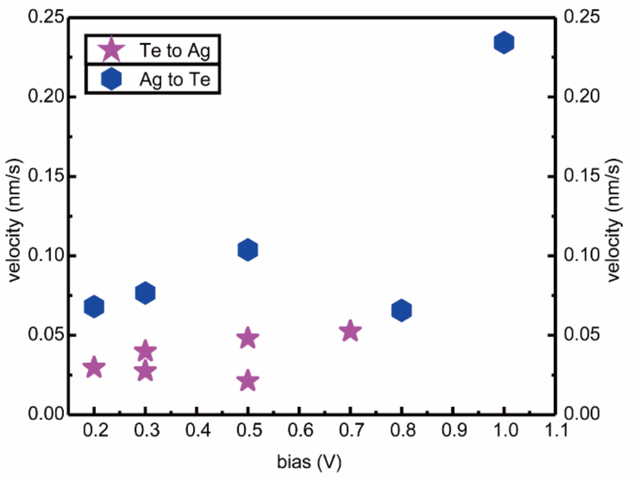

Supplementary Figure 14. The velocity of the growth of Ag_2Te layers (pentagram, when Te diffuse into Ag by surface diffusion) and the velocity of the transformation (hexagon, when Ag diffuse into Te by bulk diffusion) under different bias.

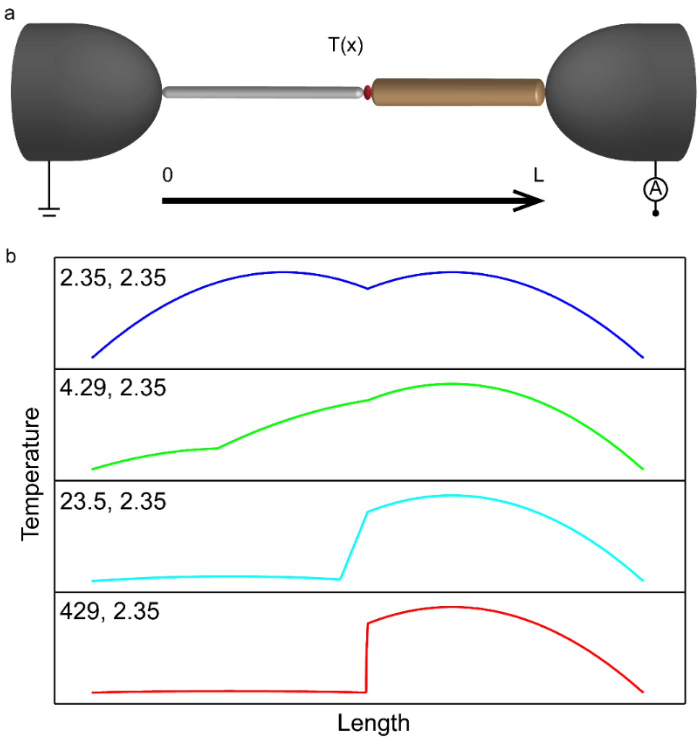

Supplementary Figure 15. The temperature distribution of the 1D conductor system. a Schematic diagram for a suspended 1D self-heating conductor (including contact resistance) between two electrodes. **b** The temperature distribution of the system for various thermal conductivity (assuming the constant electrical conductivity). The number in the upper right corner of the figure are the thermal conductivities of the nanowires. The bottom red temperature profile is corresponding to the condition of Ag and Te nanowires.

Supplementary Note 1: Analytical analysis of the temperature in a suspended 1D conductor system

When a voltage bias is applied on the 1D conductor system, heat will be generated along the conductor via Joule heating and in vacuum (in a TEM) the heat can only be dissipated along the conductor through the two contacts at it ends. For only one nanowire in the suspend conductor system, the heat transport equation is given by[2]

$$-\kappa \frac{d^2T}{dx^2} = \sigma E^2 \tag{1}$$

here, the left side is the thermal conduction and the right side refers to the thermal

energy generated by the current. T is the temperature, κ is the thermal conductivity, σ is the electrical conductivity, and E is the electric field intensity. Under constant thermal and electrical conductivity and symmetric constant temperature boundary conditions, the equilibrium temperature distribution over the 1D conductor is parabolic, which is given by

$$T(x) = -\frac{\sigma E^2}{2\kappa}x^2 + \frac{\sigma E^2 L^2}{8\kappa} + T_0 \tag{2}$$

T_0 is the boundary temperature (room temperature). The highest temperature is

$$T_{\max} = \frac{\sigma E^2 L^2}{8\kappa} + T_0 = \frac{\sigma U^2}{8\kappa} + T_0 \tag{3}$$

If the Umklapp phonon-phonon scattering process is considered, that is, κ is inversely proportional to the temperature, which is given by $\kappa = 1/\alpha T$ (α is a constant). The maximum temperature is modified to be

$$T_{\max} = T_0 \exp\left(\frac{\alpha\sigma U^2}{8}\right) \tag{4}$$

On this base, we reconsider the situation in this experiment. The 1D conductor system of Ag-Te nanowires is still in a state of thermal equilibrium. We suppose that the system can be regarded as a 1D system with length L_1 for Ag nanowire and L_2 for Te nanowire. Moreover, the contact resistance is considered to be an ideal resistance R_c without volume. The system can be regarded as the accumulation of three parts: Ag nanowire, Te nanowire, and the contact resistance. Assuming that the time required for the system to reach thermal equilibrium is t, the heat generated by R_c is given by

$$Q = I^2 R_c t \tag{5}$$

where I is the current. The heat generated by R_c can only be dissipated along the Ag and Te nanowires. Therefore, the heat conduction equation is given by

$$Q = A_1 \frac{(T - T_0)\kappa_1}{L_1} + A_2 \frac{(T - T_0)\kappa_2}{L_2} \tag{6}$$

here, A is the cross-sectional area of the Ag and Te nanowire, and κ is the thermal conductivity. According to Supplementary Equation (6), the temperature T of the contact is given by

$$T = T_0 + \frac{Q}{\frac{\kappa_1 A_1}{L_1} + \frac{\kappa_2 A_2}{L_2}} \tag{7}$$

The conduction of the heat energy generated from contact resistance depends mainly

on the thermal conductivity of the two nanowires. The temperature distribution of the 1D system could be acquired by the accumulation of the three parts: the linear temperature distribution of the contact resistance, the parabolic temperature distribution of Ag and Te nanowires.

Following this analysis, in the experiments the temperature profile along the 1D conductors can be determined for a given system with known parameters. In Ag-Te system, the thermal conductivity of Ag (429 $W \cdot m^{-1} \cdot K^{-1}$) is much larger than Te (2.35 $W \cdot m^{-1} \cdot K^{-1}$)[3], therefore Ag nanowire can be considered to be a conduit and the heat due to Joule heating is mainly generated on the contact resistance and Te nanowire. Use typical parameters for Te, the maximum temperature of Te nanowire can be estimated as around 570K with Supplementary Equation (3) or (4). The value of contact resistance is range from 10^4~10^9 Ω due to different conditions of contact. According to Supplementary Equation (5), (6), and (7), the temperature of the contact resistance in a dc biased 1D conductor system can be estimated as a range from 400K~2000K. However, applying typical parameters for Ag nanowires to Supplementary Equation (3), we can estimate the maximum temperature rise to be less than 10^{-2}~10K. The real temperature distribution of the system can be acquired by accumulation of the three parts. Notably, temperature change generated by the contact resistance on Ag and Te nanowires is linear, which is proportional to the thermal conductivity. Therefore, the temperature distribution of the Ag-Te system acquired by the analysis is shown in Supplementary Figure 13b. The side of Te nanowire will show a much higher and slowly changing temperature while the temperature reduces to the room temperature in a very short distance in Ag nanowire. The figure also shows the temperature profile in other conditions where thermal conductivities of the two nanowires are in different situations. When the difference of the thermal conductivities of the two side materials is pretty large, the difference of temperature on two side will be large too. Generally, on the side with a low thermal conductivity, the temperature generated from Joule heating will be much higher than the other side.

Therefore, we can tailor the type of the atomic diffusion in a dual-conductor system by the method and prepare different hetero-structures. If we want to synthesize a A-B core-shell structure, then we should ensure that the material B migrate in material A is by

surface diffusion. At that condition, we could set the system in a low temperature, in which the atom cannot acquire enough energy to get over the barrier of bulk diffusion.

Supplementary References

1. Zhang, Q. et al. Electrically driven cation exchange for in situ fabrication of individual nanostructures. *Nat. Commun.* **8**, 14889 (2017).
2. Huang, X. et al. Analytical analysis of heat conduction in a suspended one-dimensional object. *Appl. Phys. Lett.* **95**, 143109 (2009).
3. Cotton, A. F., Wilkinson, G., Bochmann, M. & Murillo C. A. *Advanced inorganic chemistry*. Vol. 6. (Wiley, New York, 1988).
