# Supplementary material for: Tailoring atomic diffusion for in situ fabrication of different heterostructures
Source: Nat Commun. 2021 Aug 10;12:4812. doi: 10.1038/s41467-021-25194-2 (PMC8355324; doi:10.1038/s41467-021-25194-2)
Supplement: Supplementary file 3 — Description of Additional Supplementary Files [file 41467_2021_25194_MOESM3_ESM.pdf]

Description of Additional Supplementary Files

Supplementary Movie 1. The formation evolution of Ag_2Te-Ag core-shell structure under a positive bias (Ag towards Te) of 0.3 V.

Supplementary Movie 2. In situ growth of Ag_2Te layer during the formation of core-shell structures.

Supplementary Movie 3. In situ formation of Ag_2Te-Te segmented heterostructure under a negative bias of 0.8 V. Scale bar is 50 nm.

Supplementary Movie 4. The motion of the reaction frontier during the formation of segmented heterostructures. Scale bar is 10 nm.
